# Novel heterozygous mutation in *alpha-2-macroglobulin (A2M)* suppressing the binding of amyloid-β (Aβ)

**DOI:** 10.3389/fneur.2022.1090900

**Published:** 2023-01-09

**Authors:** Guozhen Qiu, Liming Cao, Yong-Jun Chen

**Affiliations:** ^1^Cognitive Impairment Ward of Neurology Department, The Third Affiliated Hospital of Shenzhen University, Shenzhen, China; ^2^Department of Neurology, The First Affiliated Hospital of Shenzhen University, Shenzhen, China; ^3^Department of Neurology, The Affiliated Nanhua Hospital, Hengyang Medical College of University of South China, Hengyang, China

**Keywords:** Alzheimer's disease, A2M, amyloid-β, mutation, whole-exome sequencing

## Abstract

**Introduction:**

Many studies have suggested that the alpha-2-macroglobulin (*A2M*) gene may be involved in the pathogenesis of Alzheimer's disease (AD). A2M encoded by the *A2M* gene can specifically bind to the β-amyloid peptide and prevent fiber formation.

**Methods:**

The patient in this study had progressive memory loss at the age of 60 years and underwent a series of neuropsychological tests, cranial magnetic resonance imaging (MRI), cerebrospinal fluid (CSF) biomarker analysis, and whole-exome sequencing (WES) to evaluate possible mutations. We used in silico tools and three-dimensional (3D) protein structure prediction to analyze the pathogenicity of the mutation and used a co-immunoprecipitation experiment to study the effect of mutations on amyloid-β (Aβ) binding.

**Results:**

Based on neuropsychological tests, cranial MRI, and CSF biomarker analysis, the patient was diagnosed with AD. WES showed that there was a missense mutation in *A2M* (c.1229A>C, p.N410T). Bioinformatics analysis showed that this mutation was pathogenic. Moreover, 3D protein structure analysis showed that the A2M Asn410 residue was an N-glycosylation site, which was necessary for A2M activation to bind to Aβ. Missense mutations led to the loss of glycosylation at this site, which suppressed the binding of Aβ. The functional experiment also confirmed the prediction: the interaction between A2M and Aβ from the patient's plasma was weakened.

**Conclusions:**

Our results demonstrate that this novel A2M p.N410T mutation may have a pathogenic role in AD, by altering the binding interactions between A2M and Aβ.

## Introduction

Alzheimer's disease (AD) is a neurodegenerative disease characterized by progressive cognitive impairment associated with a reduction in daily activities (1). The *APP* gene encodes the amyloid-β (Aβ) precursor protein, which is cleaved by β-secretase and γ-secretase complex to produce Aβ (2). A disturbance in Aβ production and clearance plays a crucial role in the progression of AD (3).

The *A2M* gene encodes alpha-2-macroglobulin and is located on chromosome 12. A2M specifically binds to Aβ peptide (major component of Aβ) to prevent the formation of fibrils (4) and attenuates neurotoxicity of Aβ (5), and it also mediates the degradation and clearance of Aβ (6, 7). Therefore, these findings suggest that A2M may be involved in the AD pathological pathway. It has been reported that two common polymorphisms in the *A2M* gene are associated with AD development. One is a 5-bp (CCATA) insertion/deletion (5 bp I/D) at the 5′-splice site of exon 18 (8). It is known that exon 18 encodes the bait domain of A2M, which is responsible for the activation of A2M (9). The other is a A/G transition which causes an isoleucine (Ile) to valine (Val) exchange at codon 1000 (Ile1000Val) (10), and this site is near the C-terminal region of A2M which inhibits the β-sheet formation and fibril-formation activities of Aβ (4).

In this study, a Chinese patient with early-onset AD was recruited for genetic analysis. A new missense heterozygous *A2M* mutation (chr12-9256872, c.1229A>C, p.Asn410Thr) was identified *via* whole-exome sequencing (WES). We used *in silico* tools and three-dimensional (3D) protein structure prediction to analyze the pathogenic mechanism of the mutation site. An *in vitro* study was performed to evaluate the effects of mutation on Aβ binding.

## Materials and methods

### Subject

The patient was treated in the memory clinic of the Third Affiliated Hospital of Shenzhen University, Guangdong, China. The diagnosis of AD was based on the 2018 National Institute on Aging and Alzheimer's Association (NIA-AA) research framework (11). The patient's cognitive status was evaluated using the Mini-Mental State Examination (MMSE). The patient also underwent 1.5T structural Magnetic Resonance Imaging (MRI), [^11^C]-Pittsburgh compound B (PIB)-PET and [^18^F] fluoro-2-deoxyglucose (FDG)-PET scans. Analysis of cerebrospinal fluid (CSF) biomarkers was performed by the Oumeng V-Medical Laboratory (Hangzhou, China). The ethics committee of the Third Affiliated Hospital of Shenzhen University approved this study and a written informed consent was obtained from the participant and his legal guardian.

### Genetic testing and *in silico* analysis

DNA peripheral blood extraction was performed using CWE2100 Blood DNA Kit V2 (CWBiotech, China, CW2553). WES was conducted by Running-Gene Inc. (Beijing, China) to identify potential disease-causing gene variants. Exome capture was performed using the SureSelect Human All Exon Kit (Agilent Technologies, USA), and high-throughput sequencing was performed using the Illumina Novaseq 6000 platform (Illumina, USA). The raw exome sequencing data were analyzed by bioinformatics analysts from Running-Gene Inc. The Genome Analysis ToolKit (GATK, www.broadinstitute.org/gatk) was used to call single nucleotide variant and the variant was annotated by ANNOVAR (http://annovar.openbioinformatics.org/en/latest/). Sanger sequencing was conducted to validate the candidate variants found in WES. The apolipoprotein E (*APOE*) genotype was obtained by Sanger sequencing.

Population databases including NHLBI Exome Sequencing Project 6500 (ESP6500) (https://evs.gs.washington.edu/EVS/), 1000 Genomes database (https://www.ncbi.nlm.nih.gov/variation/tools/1000genomes/) and Genome Aggregation Database (GnomAD) (http://gnomad.broadinstitute.org) were searched to determine the minor allele frequency (MAF) of A2M N410T.

The pathogenicity prediction of the variants was performed using SIFT (12), and PROVEAN (13). The thermal stabilities of protein structures for the missense mutations were assessed by DUET (14). Evolutionary conservation of the mutation site was analyzed and visualized in UGENE using ClustalW (15). The crystal structure of the protein was downloaded from the Protein Data Bank (PDB, www.rcsb.org), and the effect of the mutations was evaluated using PyMol (PyMol Molecular Graphics System, version 2.3.0, Schrodinger LLC). The final variant classifications were based on the American College of Medical Genetics and Genomics (ACMG) 2015 criteria (16).

### Co-immunoprecipitation and immunoblot analysis of Aβ-binding to A2M

Plasma was collected from the patient and a healthy control (with A2M wild type [WT] and had no evidence of dementia, cerebrovascular disease, or head injury). A2M was purified from plasma according to previously described method (17). Methylamine treatment of A2M was performed by dialyzing against 200 mM methylamine in 50 mM Tris buffer (pH 8.2). Methylamine-treated A2M samples were incubated with Aβ40-HIS/Aβ42-HIS (Yuan Peptide, Nanjing, China). Co-immunoprecipitation (CoIP) was performed according to the instructions of the Pierce cross-linking CoIP kit (Thermo Fisher Scientific, USA). In brief, Pierce Protein A/G Magnetic Beads were incubated with HIS-tag antibody (ab18184, Abcam) for 4 h or IgG (MG115, Invitrogen) as a negative control and subsequently with the protein sample overnight at 4°C. Eluted CoIP lysates were separated using SDS/PAGE and transferred to polyvinylidene difluoride (PVDF) membranes (Millipore). Membranes were blocked overnight followed by incubation with anti-A2M antibody (ab133299, Abcam) or anti-Aβ (ab180956, Abcam). After washing, the blots were incubated with horseradish peroxidase-conjugated secondary antibody, and visualized using Pierce ECL Western (Thermo Fisher Scientific). The levels of CoIP were determined by measuring bands intensity with Quantity One software (Bio-Rad, Hercules, CA). The quantitative results presented are the mean of three independent experiments. Statistical significance was determined using the unpaired student *t*-test.

## Results

### Index patient clinical description

**Figure 1:**
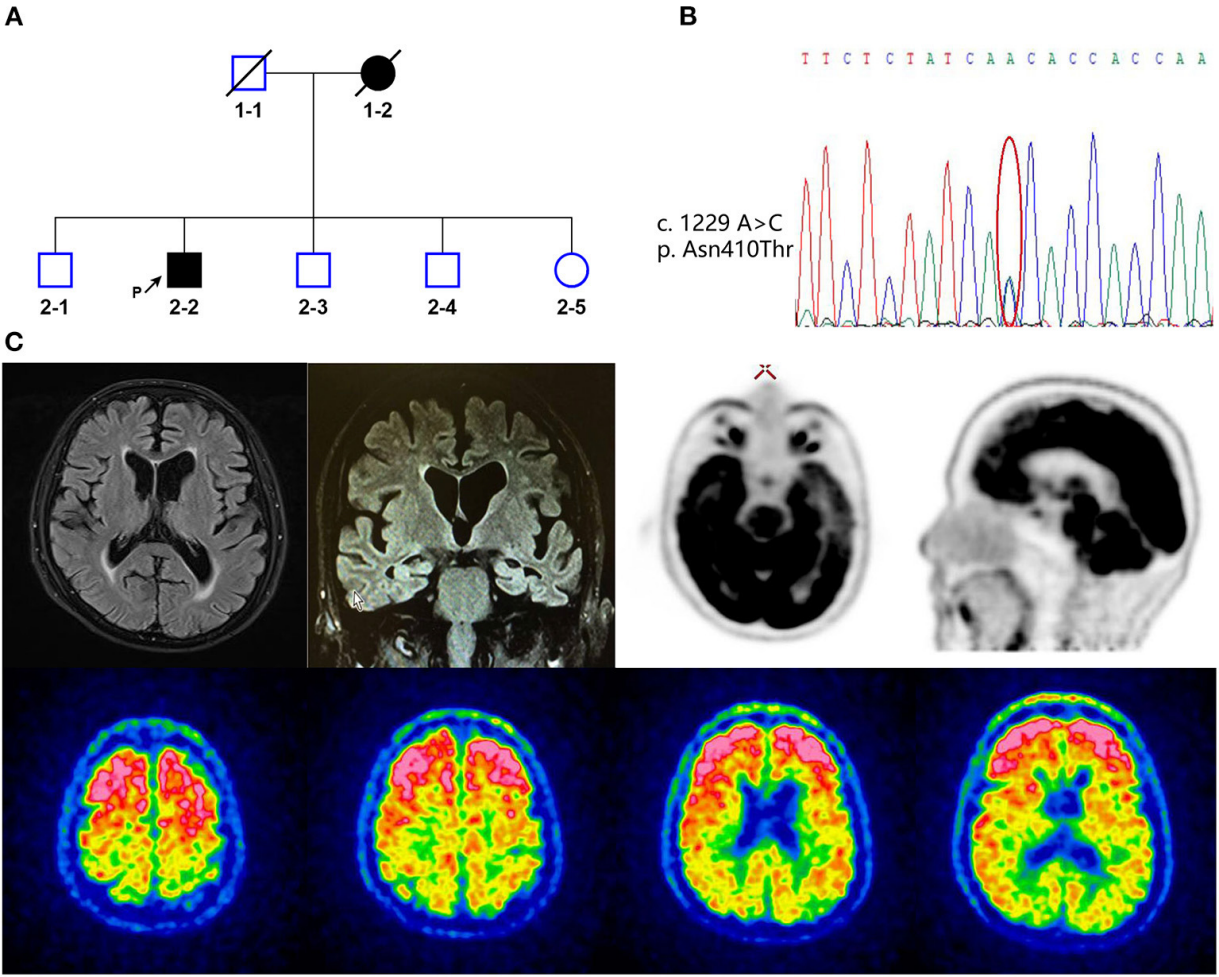
**(A)** Family pedigree of the patient with *alpha-2-macroglobulin* (A2M) p.Asn410Thr (2-2). Black arrow indicates the proband. Square indicates male, circle indicates female, and slashes denote deceased members. Open squares and circles mean asymptomatic family members, who were not diagnosed with disease. Filled squares and circles indicate patient. **(B)** Whole-exome sequencing data of *A2M* p.Asn410Thr (c.1229A>C) mutation. **(C)** Representative MRI FLAIR weighted (up-left), FDG-PET (up-right), and PIB-PET images (bottom) of the patient.

A 65-year-old male retired university professor presented with 5 years of gradually progressive memory loss and behavioral changes. A family history indicated dementia in his mother (Figure 1A). At the age of 60 years, the proband complained of difficulty in concentration and delayed thinking. He then gradually developed memory loss (forgetting recently learned information) and returned to the memory clinic at the age of 64 years. The patient was diagnosed with mild cognitive impairment, and the patient began treatment with 2.5 mg of donepezil per day, but his cognitive condition continued to deteriorate. He could not recognize his family members and had repetitive questioning, difficulty in word-finding, persecutory ideas and delusions about food (believing food is poisoned). He lost interest in his daily activities, including eating, and was unable to take care of himself in daily life. He also developed behavioral and psychological symptoms. He sometimes showed excitement, rambling, self-talking, suspicion, irritability, and swearing and sometimes showed apathy, emotional blunting, and limited speech. On examination, the patient demonstrated delayed responses; impaired orientation to time and place; impaired memory recall; impaired attention/calculation; and impaired language comprehension. He scored 11/30 on the MMSE (cut-off < 24). The patient's *APOE* genotype was ε3/ε3. A brain MRI scan showed symmetrical atrophy in the bilateral frontal, temporal, and parietal cortices and hippocampal area (Figure 1C). [^11^C]PIB-PET revealed increased amyloid accumulation predominantly in the frontal lobe; parietal, temporal, and occipital lobes; and anterior and posterior cingulate gyrus (Figure 1C). [^18^F]FDG-PET indicated significant hypometabolism in the left lateral temporal and left medial frontoparietal lobes (Figure 1C). CSF showed a decreased Aβ42 level (172 pg/mL, normal values >651 pg/mL), increased T-tau (376 pg/mL, normal values < 290 pg/mL), and P-tau (68 pg/mL, normal values < 61 pg/mL). All these findings clearly supported the diagnosis of AD.

### Genetic analysis and variant interpretation

**Figure 2:**
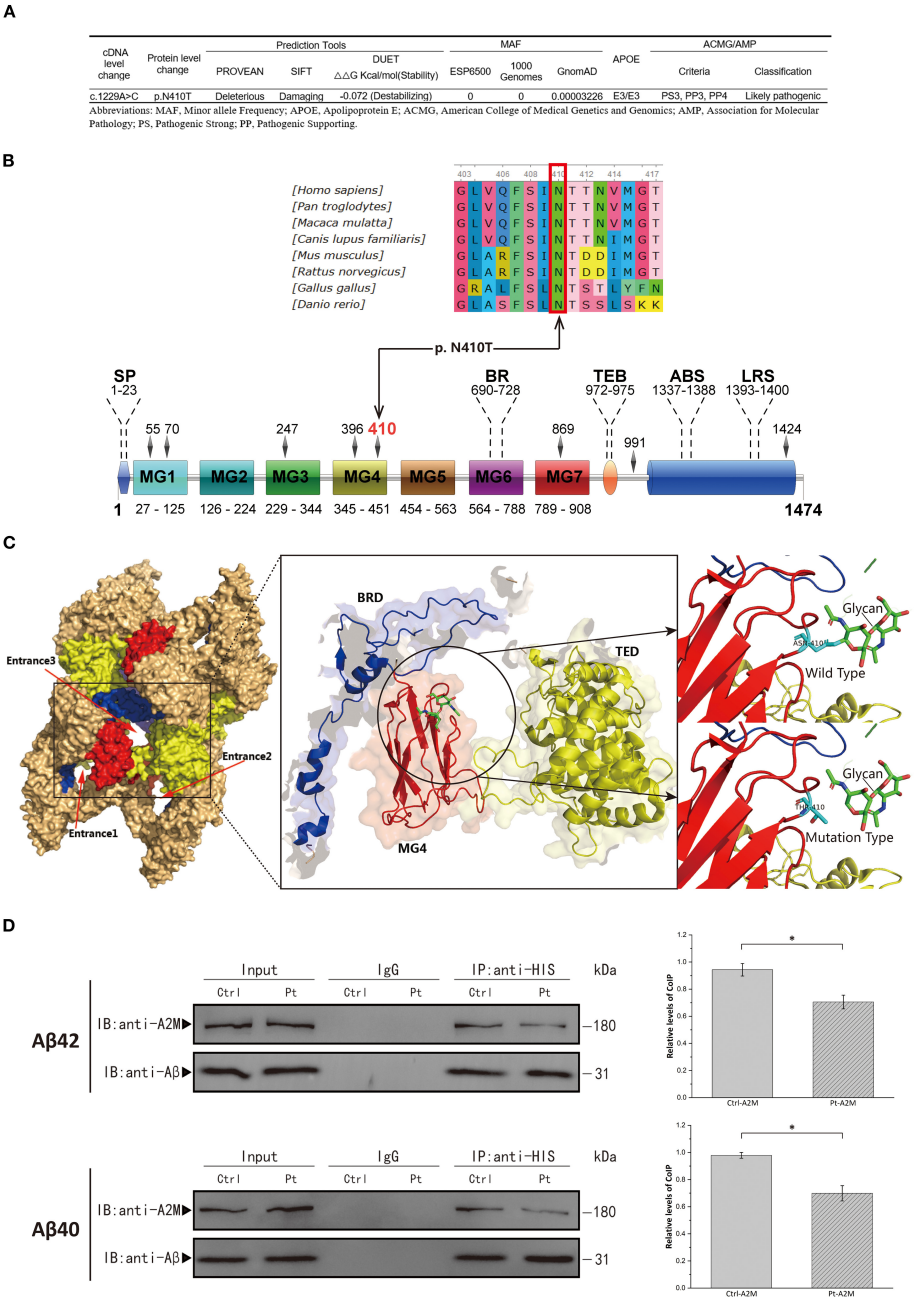
**(A)** Schematic of p.Asn410Thr mutation identified in the *A2M* gene. **(B)** Upper, conservation analysis of the mutation site. Amino acids marked with red column are highly conserved among all shown species. Lower, schematic representation of the full-length A2M protein; SP, signal peptide; BR, bait region; TEB, thiolester bond; ABS, Aβ binding site; LRS, LRP recognition site. **(C)** Conformational prediction of p.Asn410Thr mutation. TED, thiolester domain; BRD, bait region domain; MG, macroglobulin-like domain. **(D)** Left Panel, Co-immunoprecipitation (CoIP) analysis of interaction of Aβ40/Aβ42 with plasma A2M samples. Ctrl, healthy control; Pt, patient. Right Panel, Quantitative analysis of the CoIP is presented in the graph representing the relative amounts of coimmunoprecipitated protein compared with the immunoprecipitated bait. All the data are presented as mean ± SEM, *n* = 3, *p < 0.05.

The WES verified by Sanger sequencing revealed a heterozygous nucleotide substitution in exon 11 of the *A2M* gene (c.1229A>C, p.Asn410Thr) (Figure 1B). Genetic analysis data were not available for the proband's other family members. This variant had not previously been reported and was not discovered in the ESP6500, 1000 Genomes, but extremely low frequency was found in GnomAD database (Figure 2A). It caused a change in the amino acid that was highly conserved throughout vertebrates (Figure 2B). It was predicted to be “Deleterious” by PROVEAN and was described as “Damaging” by SIFT. The structure-based tool DUET also predicted p.N410T as “destabilizing” (Figure 2A). A schematic representation of the full-length A2M protein showed that p.N410T was located in the N-glycosylation site of the macroglobulin-like-4 (MG4) domain (Figure 2B). Structure prediction of WT and mutation type revealed the loss of glycosylation at 410 site (Figure 2C).

### Effects of alpha-2-macroglobulin (A2M) Asn410Thr on the binding of amyloid-β to A2M

Aβ40/Aβ42 binding to patient and healthy control plasma A2M was determined by CoIP analysis. After the plasma samples were treated with methylamine to induce conformational change in A2M, the A2M in the patient sample bound less Aβ40/Aβ42 than the A2M in the control sample (Figure 2D), which ascertained the pathogenicity of the novel mutation (p.Asn410Thr).

## Discussion

In this study, a novel mutation, p.Asn410Thr was reported in A2M in a Chinese patient with a family history of dementia, who presented with progressive short-term memory loss and behavioral changes. The atrophy patterns observed on MRI and the findings of FDG, PIB-PET, and CSF biomarkers provided evidence for the presence of AD pathology. In fact, no other pathogenic mutations at A2M codon 410 have been reported in the literatures. c.1229A>C has not been discovered in the ESP6500 and 1000 Genomes, but extremely low frequency was found in GnomAD database, indicating that it is significantly rare in normal populations. The structure-based approach (PROVEAN) and the evolutionary-based approach (SIFT) revealed p. N410T as a damaging variant. Further, the destabilizing effect of the mutation on protein stability was also confirmed by the DUET server. Through *in vitro* experiments, we demonstrated that the binding of Aβ to A2M in the patient sample was less than that in the healthy control.

The A2M Asn410 residue is an N-glycosylation site, where a glycan is covalently attached to asparagine (Asn) by an N-glycosidic bond (18). It is known that N-glycans affect many properties of glycoproteins including their conformation, solubility, and activity (19). Previous studies have also revealed that changes in the glycosylation of A2M are associated with systemic lupus erythematosus and multiple sclerosis (17, 20). The N-glycosylation site identified in this patient was located in the MG4 domain. The tetramerization of each subunit formed three entrances to the central cavity of the A2M tetramer. These entrances were essential for the binding of noncovalent ligands (i.e., Aβ) to the central cavity, of which entrance 3 was framed by the MG4 domain, thiolester domain (TED) and bait region domain (BRD). Access to entrance 3 was modulated by the glycan chains attached to N410 (21). The loss of glycosylation at the 410 site may affect the conformation of entrance 3.

Based on the above findings, we interpret this variant to be likely pathogenic, according to the American College of Medical Genetics and Genomics (ACMG) guideline, including (1) pathogenic strong (PS)3, well-established *in vitro* or *in vivo* functional studies supportive of a damaging effect on the gene or gene product; (2) pathogenic supporting (PP)3, multiple lines of computational evidence supporting a deleterious effect on the gene or gene product; and (3) PP4, patient's phenotype or family history is highly specific for a disease with a single genetic etiology.

## Conclusions

A novel *A2M* heterozygous mutation c.1229A>C (p.Asn410Thr) was identified in a Chinese male patient with early-onset AD. This mutation was located at an N-glycosylation site, which might result in an altered affinity for A2M binding to Aβ. According to the ACMG criteria, the mutation may be a likely pathogenic variant. Limitation of this study was that the segregation analysis could not be performed, because all living relatives of the patient declined genetic testing for concern about genetic discrimination. In the future, much more additional functional studies should be needed to verify.

## Data availability statement

The datasets presented in this article are not readily available because of ethical and privacy restrictions. Requests to access the datasets should be directed to the corresponding author.

## Ethics statement

The studies involving human participants were reviewed and approved by the Ethics Committee of The Third Affiliated Hospital of Shenzhen University. The patients/participants provided their written informed consent to participate in this study.

## Author contributions

GQ: conceptualization, methodology, data curation, visualization, and writing—original draft. LC: visualization and investigation. Y-JC: supervision and writing—review and editing. All authors contributed to the article and approved the submitted version.
